# Measurement of muscle health in aging

**DOI:** 10.1007/s10522-017-9697-5

**Published:** 2017-04-04

**Authors:** Peter Francis, Mark Lyons, Mathew Piasecki, Jamie Mc Phee, Karen Hind, Philip Jakeman

**Affiliations:** 1 Musculoskeletal Health Research Group, School of Clinical and Applied Sciences, Leeds Beckett University, Leeds, LS13HE UK; 2 Human Science Research Unit, Center for Intervention in Inflammation, Infection and Immunity, University of Limerick, Limerick, Ireland; 3 School of Healthcare Science, Manchester Metropolitan University, Manchester, UK; 4 Carnegie Research Institute, Leeds Beckett University, Leeds, UK

**Keywords:** Healthy aging, Sarcopenia, Muscle strength, Functional capability

## Abstract

Muscle health is a critical component in the struggle against physical frailty and the efforts to maintain metabolic health until the limit of chronological age. Consensus opinion is to evaluate muscle health in terms of muscle mass, strength and functional capability. There has been considerable variability in the components of muscle health which have been investigated in addition to variability in the tools of assessment and protocol for measurement. This is in stark contrast to the validated measurement of bone health across the adult life span. The purpose of this review was to identify indices of muscle mass, strength and functional capability most responsive to change with ageing and where possible to provide an estimate of the rate of change. We suggest lean tissue mass (LTM) or skeletal muscle (SM) is best evaluated from the thigh region due to its greater responsiveness to ageing compared to the whole body. The anterior compartment of the thigh region undergoes a preferential age-related decline in SM and force generating capacity. Therefore, we suggest that knee extensor torque is measured to represent the force generating capacity of the thigh and subsequently, to express muscle quality (strength per unit tissue). Finally, we suggest measures of functional capability which allow participants perform to a greater maximum are most appropriate to track age-related difference in functional capacity across the adult lifespan. This is due to their ability encompass a broad spectrum of abilities. This review suggests indices of muscular health for which reference ranges can be generated across the lifespan.

## Background

Life expectancy in Europe is set to increase by 8.5 and 6.9 years for men (84.5 years) and women (89.0 years) respectively by the year 2060. However, up until 2009, ‘healthy’ life expectancy in Europe lagged two decades behind chronological age (Rechel et al. 2013). Old age at best leads to compromised physical prowess (Drey et al. 2016) and at worst can lead to a crippling loss of independence (Wang et al. 2013), often accompanied by metabolic disease (Leenders et al. 2011). Muscle health is therefore a critical component in the struggle against physical frailty and the efforts to maintain metabolic health until the limit of chronological age. Sarcopenia comes from the Greek words ‘Sarx’ meaning flesh and ‘penia’ meaning loss and has been used to describe the age related decline in muscle mass (Rosenberg 1989). Many authors since (Morley et al. 2001; Cruz-Jentoft et al. 2010), have used sarcopenia as an all-encompassing term, to describe the accompanying loss of strength and functional capability with age.

Originally, the decline in muscular strength was thought to be caused by a loss of muscle mass (Frontera et al. 1991). In an approach similar to the measurement of bone health, researchers focused on developing diagnostic criteria for muscle health based on classifying individuals as having high or low indices of muscle mass relative to a healthy young adult norm (Gallagher et al. 1997; Baumgartner et al. 1998; Janssen et al. 2000). There is now a growing body of evidence spanning over 3 decades (Larsson et al. 1979; Clarkson et al. 1981), that suggests muscle mass and strength are not as closely linked as previously assumed (Clark and Manini 2008; Manini and Clark 2012). Prior to a change in absolute mass or cross-sectional area (CSA), muscle undergoes a series of physiological changes with ageing that are implicit in a decrease in strength. There is a reduction in the number of motor units and a resultant increase in the size of motor units because of the compensatory collateral sprouting by surviving neurons (Vandervoort 2002; Piasecki et al.2016a, b). Furthermore, maximal motor unit firing rates are reported to be 35–40% lower than young adults (Kamen et al. 1995) and exhibit greater variability in motor unit discharge (Christou 2011). Subsequently, at the tissue level the excitation–contraction coupling processes are thought to be impaired due to impairments in calcium release from the sarcoplasmic reticulum. These changes are compounded by an increase in inter and intramuscular adipocyte content (Delmonico et al. 2009) that is thought to directly impair cross-bridge kinetics (D’Antona et al. 2003). As a result of the increase in fat infiltration and connective tissue, the net contractile mass is less. In fact, non-contractile mass can account for 15% of total muscle CSA, an estimate that is 2.5 fold greater than in young controls (Taaffe et al. 2009).

Consequently, there is a growing interest in the measurement of age-related change in muscle performance rather than size alone. As a result, the consensus in Europe (Cruz-Jentoft et al. 2010) and America (Fielding et al. 2011) among expert working groups is that muscle health should be assessed in terms of muscle mass, muscle strength and functional capability. Low relative skeletal muscle (SM) mass has been shown to be associated with functional impairment (Janssen et al. 2002) measured by the short physical performance battery (SPPB) (Guralnik et al. 1994). Increasing knee extensor torque has been associated with improved walking speed and the ability to rise from a chair (Ostchega et al. 2004; Hairi et al. 2010). Knee extensor speed of contraction has been found to be predictive of gait speed in mobility limited older adults (Sayers et al. 2005). Despite established relationships between muscle mass, strength and functional capability in mobility limited older adults, comparatively little is known about the time course and transition to functional impairment in healthy older (>50 y) adults (Murphy et al. 2014). Furthermore, there has been considerable variability in the components of muscle mass, strength and functional capability which have been investigated in addition to variability in the tools of assessment and protocol for measurement. This is in stark contrast to the validated measurement of bone health across the adult life span (Kanis et al. 1994). The focus of this review is not to discuss the mechanistic causes of functional impairment but to identify indices of muscle mass, strength and functional capability most responsive to change with ageing and where possible to provide an estimate of the rate of change.

## Age-related change in muscle or lean tissue mass

Currently, imaging methods including magnetic resonance imaging (MRI) and computed tomography (CT) represent the accepted criterion method for quantifying whole body and regional skeletal muscle (SM). This is due to their ability to distinguish between fat, skeletal muscle and other non-muscle fat-free components such as connective tissue (Wang et al. 1996; Mitsiopoulos et al. 1998; Levine et al. 2000). Dual energy x-ray absorptiometry (DXA), more commonly used in the assessment of bone health has been found to be a reliable cost effective method of quantifying whole body and regional non-osseous lean tissue mass (LTM) with a low radiation dose (Wang et al. 1996; Levine et al. 2000). Despite a strong correlation between CT and DXA in the estimation of SM (r = 0.88, P < 0.001), reporting the age-related decline in SM using DXA requires caution as it has been shown to overestimate whole body and regional SM. This methodological issue may mask age or therapeutic related changes in SM (Delmonico et al. 2008; Nilwik et al. 2013; Maden-Wilkinson et al. 2013).

Numerous studies have attempted to quantify the rate of decline in SM as though it is a uniform process which begins at the completion of growth (Gallagher et al. 1997; Silva et al. 2010). The suggestion that LTM begins to decline in the third decade stems from studies evaluating the decline in skeletal mass relative to body mass which produces an inflated decline due to an increase in fat mass (Janssen et al. 2002). Janssen et al. (2000) suggest age is not associated with appendicular SM, as measured by MRI, until after ~45 y. Furthermore, changes in whole body LTM as measured by DXA or hydro-densitometry, albeit less sensitive measures of SM, are subtle enough not to be detected until after 60 y in either cross-sectional (Kyle et al. 2001) or longitudinal (Hughes et al. 2002) analysis. Estimating the age-related decline in SM across the adult lifespan is difficult due to incomplete data sets across age ranges which have led to researchers using equations to predict declines (Gallagher et al. 1997; Baumgartner et al. 1998). This has been compounded by existing literature containing multiple ethnic groups. There is considerable variability in the age-related decline in SM amongst Hispanics, African-American, Caucasian, and Asians. The rate of change in SM in these ethnic groups differs between men and women (Baumgartner et al. 1998; Janssen et al. 2002; Silva et al. 2010). Therefore, it is recommended age-related change in SM is reported according to gender and ethnicity.

A 1–2% decline in SM after age ~50 y is often quoted in review papers on the topic of sarcopenia (Thomas 2007; Rolland et al. 2008). In the section below, we intend to demonstrate from the available data that the estimate at the whole body is actually far more conservative. Furthermore, we suggest the rate of decline in SM for middle-aged (40–70 y) and older (>70 y) adults are not uniform nor are the rates of decline at the whole body, appendicular or limb specific regions of the body.

Frontera et al. (1991) reported stature (ht2) corrected fat free mass (FFM; hydrostatic weighing) in older (45–78 y) adults to decline at a rate of 3.4 and 3.1% per decade men and women respectively from a cross sectional analysis. The per decade decline from peak levels of SM is greater in men (4.2%) and women (3.8%) when estimated using MRI (Janssen et al. 2000); perhaps due to the methodological differences discussed above. These cross-sectional analysis which suggest a rate of decline between 3.1 and 4.2% per decade are in agreement with consensus among recent reviewers (Narici and Maffulli 2010; Mitchell et al. 2012) who report that healthy adult LTM declines ~20–28% between the 2nd and 8th decades of life (3.3–4.6% per decade). Mitchell et al. (2012) reviewed studies using indirect (total body potassium, 24 h urinary creatinine excretion) and direct methods (MRI, CT, DXA) of assessing lean body mass between the years of 1972 and 2011. The median decline per decade was 4.7 and 3.7% in men and women respectively. This decline becomes identifiable at ~45 y (Janssen et al. 2000) but is not statistically different from a young adult until ~50 y (Janssen et al. 2000; Kyle et al. 2001; Hughes et al. 2002). Considering the evidence above, a 0.3–0.5% per annum decline in SM may be considered a more realistic estimate of the change in SM as a result of normal ageing than the 1–2% widely reported.

### Site-specific changes in muscle or lean tissue mass 

Appendicular SM is the main proponent of functional capability. More specifically, the lower limbs possess the major muscles involved in locomotion such as walking, rising from a chair and stair climbing. Therefore, it has been postulated that appendicular or limb specific SM changes may be of more functional significance. Lynch et al. (1999), as part of the Baltimore Longitudinal Study of Aging (BLSA), reported women to have a greater rate of decline in leg LTM (DXA) than men between the 4th and 7th decade (4.9 vs. 2.6% per decade). These findings were extended by Janssen et al. (2000) who reported women to have a greater rate of SM (MRI) decline in the lower extremities in comparison to men (5.7 vs. 3.5%). More recently, although not statistically significant, we reported mean age-related difference in LTM (DXA) between the 6th and 7th decade of life to be greater at the upper leg compared to the whole body (5.3 vs. 1.8%) in healthy women (Francis et al. 2016b). Furthermore, our research group reported upper leg LTM but not whole body LTM to increase following 12 weeks of progressive resistance training (Francis et al. 2016a). The results of these studies, in women at least, suggest lower limb LTM is a more sensitive index of age-related change in SM between the 4th and 7th decade.

Perhaps the most robust analysis to date of age-related change in muscle mass, strength and quality was reported by the Healthy Aging and Body Composition (HABC) study (Newman et al. 2003; Goodpaster et al. 2006; Delmonico et al. 2009) when describing adults in the 8th decade of life (n = 3075). The HABC study measures lower limb muscle or lean tissue mass via CT (thigh muscle area) and DXA (total leg LTM); lower limb strength via isokinetic knee extensor torque and muscle quality by expressing strength per thigh muscle area (N m/cm^2^) or total leg lean tissue (N m/kg). For the purpose of this review we discuss the rate (per decade) of decline in muscle health in cross-sectional and longitudinal analysis from the HABC study. In the case of longitudinal analysis, to provide the rate (per decade) of decline it is necessary to pool all ethnic male and all ethnic female data and divide the percentage changes by the number of years (3 or 5 years) participants were followed.

Newman et al. (2003) reported leg LTM to decline at a rate of 9–10% per decade during a cross-sectional analysis of the 8th decade; almost double the rate described in earlier decades. Three and 5 year follow up studies of older adults, ~72 years of age at baseline, revealed a 9.8–11.7% per decade decline in lower limb thigh muscle or lean tissue in men and a 6.4–9.0% decline in women (Goodpaster et al. 2006; Delmonico et al. 2009). The results of the HABC study indicate a greater rate of decline in lower limb SM or LTM in those >70 y and unlike existing data prior to 70 y, men consistently lose more LTM in comparison to women.

Within the lower limb, the anterior compartment (knee extensors) of the thigh accounts for a greater proportion of SM than the posterior compartment (knee flexors). Frontera et al. (2008) reported total thigh muscle area to decline 0.5% per annum during a 9 year follow up of adults (n = 12) in the 8th decade of life. The decline was largely dominated by the anterior compartment relative to the posterior compartment (5.7 vs. 3.2%). Further support for the preferential decline in SM of the anterior compartment has been provided with more recent evidence. Ogawa et al. (2012) reported age to be inversely associated with quadriceps femoris but not hamstring thickness (Ultrasound; US) in women and Maden-Wilkinson et al. (2013) reported quadriceps and hamstring SM to be 30% and 18% lower in older (~72 y, n = 53) adults compared to their younger (~22 y, n = 38) counterparts. We suggest that lower limb LTM as measured by DXA or quadriceps femoris SM as measured by MRI, CT or US are the most responsive indices of SM to ageing and given their association with functional capability are worthy of being used to generate reference ranges.

## Age-related change in muscle strength

Isokinetic dynamometry is the most common method of assessing voluntary strength in the appendicular regions (Arnold et al. 1993; Li et al. 1996; Lund et al. 2005; Maffiuletti et al. 2007). Strength in the arms and legs reach peak values between 25 and 35 y (Asmussen and Heebollnielsen 1962), plateau or decline slightly in those >40 y (Lindle et al. 1997; Metter et al. 1997), show definite declines in those >50 y (Lynch et al. 1999) and more rapid declines in those >65 y (Newman et al. 2003). Cross sectional and longitudinal observations of age-related change in strength vary between 8 and 15% per decade (Hurley 1995; Metter et al. 1999) in those up to ~70 y. It is generally accepted that the age-related decline in strength is greater in longitudinal compared to cross sectional observations (Clement 1974; Metter et al. 1997; Hughes et al. 2001; Goodpaster et al. 2006). Men lose strength almost evenly between the upper and lower extremities and at a greater rate than women. However, women have a greater rate of decline in the lower extremities relative to their upper extremities (Lynch et al. 1999; Hughes et al. 2001).

Upper leg (knee extensor and flexors combined) torque determined in cross-sectional and longitudinal studies declines at a rate of 8–14% per decade from peak levels up until age 70 y (Frontera et al. 1991; Lynch et al. 1999; Francis et al. 2016b). These estimates of age-related difference in maximal strength are consistent despite differences in sample size, protocol for isokinetic assessment and method for reporting the decline (mean difference vs. regression). Consistent with the preferential atrophy of SM in the anterior compartment, the knee extensors account for the majority of torque decline (Frontera et al. 2008; Francis et al. 2016b). For adults in the 8th decade of life the cross-sectional differences in knee extensor torque increase to 19–22% per decade and up to ~27–38% during longitudinal analysis (Newman et al. 2003; Goodpaster et al. 2006; Delmonico et al. 2009). The age-related change in knee extensor strength observed in the HABC study is 2–5 times greater than the SM loss in the thigh region.

## Age-related change in muscle quality

Declines in grip and knee extensor strength have been shown to occur independent of changes in limb circumference, anthropometrically determined lean body mass and thigh CSA, determined by CT (Larsson et al. 1979; MacLennan et al. 1980; Kallman et al. 1990; Overend et al. 1992). Recent evidence, discussed above, has had the benefit of modern imaging techniques and commercially available dynamometers to accurately quantify muscle mass and strength. This has served mainly to reaffirm the findings of Larsson et al. (1979) and others reported above. Many of these studies indicate that the loss of strength is somewhat greater than loss of muscle mass with aging (Hughes et al. 2001; Goodpaster et al. 2006; Delmonico et al. 2009) implying that muscle quality may be reduced. The quality of functional SM or LTM can be expressed as strength per unit of tissue. Valid and reliable measurements of segmental SM or LTM combined with measures of muscle function e.g. maximal voluntary strength, allows for the development of an appropriate index of muscle quality It is suggested that muscle quality may be able to better distinguish between those with high and low functional capability (Cruz-Jentoft et al. 2010; Hairi et al. 2010).

**Fig. 1:**
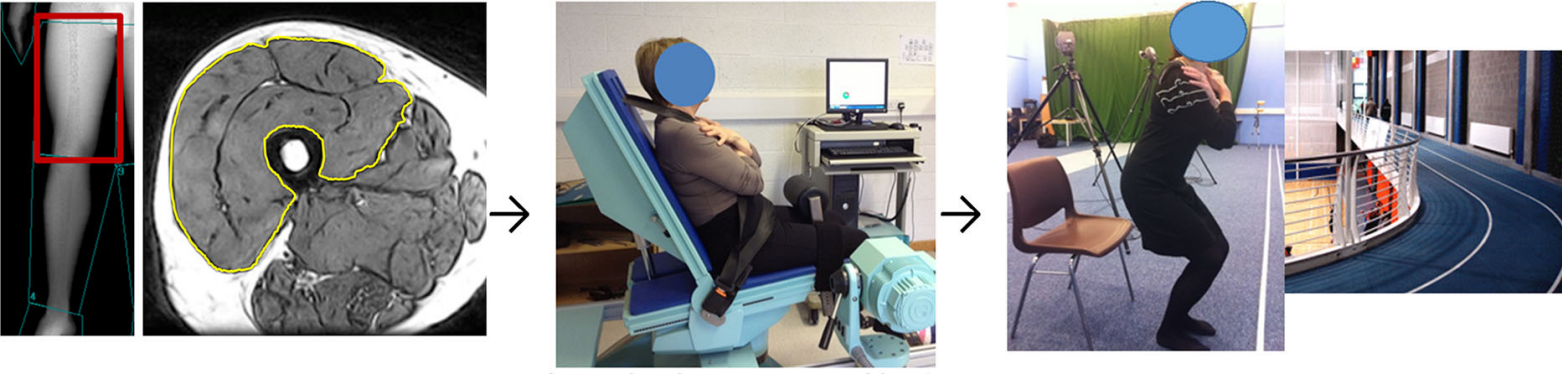
Upper leg lean tissue mass (iDXA; University of Limerick Body Composition Study) and quadriceps skeletal mass (MRI; Manchester Metropolitan Healthy Ageing Study), knee extensor torque (isokinetic dynamometry; University of Limerick Healthy Ageing Study), extended chair rise and gait speed tests (University of Limerick Healthy Ageing Study) appear to represent the indices of lower extremity muscle mass, strength and function responsive to age-related change and therapeutic intervention

Since 1992, the BLSA has measured peak torque (0–30º/s) of the arms and legs and non-osseous LTM (DXA) (Lindle et al. 1997; Lynch et al. 1999). Muscle strength was reported to have a greater rate of decline than LTM, this difference began aged ~50 y and increased with age. There was an age associated linear decline when muscle quality was expressed as knee extensor torque per CSA or LTM (Metter et al. 1999). However, the definition of muscle quality is strength per unit LTM and therefore, in theory, it would seem more appropriate to express muscle quality as upper leg (combined knee extensor and flexor) strength per unit upper leg LTM. To this aim, Lynch et al. (1999) and Francis et al. (2016b) have expressed the combined upper leg torque per kg of total and upper leg LTM respectively. Using this index the decline in men was 5.1% (Lynch et al. 1999) and ~8–10% per decade in women (Madsen et al. 1997; Francis et al. 2016b). Despite the definition of muscle quality many authors have chosen to represent muscle quality using only knee extensor torque per total upper leg SM or LTM. We have previously reported that the index of muscle quality becomes more variable when the knee flexors are included and that knee extensor torque explained a greater proportion of the variance in the combined measure. These explanations may explain the bias in the literature toward using knee extensor strength only when generating indices of upper leg muscle quality. In light of these measurement considerations; the preferential decline in knee extensor SM and strength relative to the knee flexors; and the fact that the knee extensors are used in power activities that are usually sustained across the lifespan such as climbing stairs; we suggest that the most appropriate index of muscle quality is knee extensor torque per unit SM or LTM (Fig. 1).

The HABC study quantifies muscle quality directly, based on the density of muscle obtained using CT images, and indirectly, by dividing knee extensor torque by muscle mass or volume similar to the definitions above. Goodpaster et al. (2001) reported lower muscle attenuation values (density) with increasing age, body mass index (BMI) and body fat percentage. Although advancing age and obesity were associated with reduced muscle density, the opposite effects were seen with respect to muscle size. Multiple regression analysis showed that muscle attenuation was associated with knee extensor torque (60º/s) after accounting for CSA. As muscle attenuation values increased so did muscle quality (N m/cm^2^), furthermore, men and women with the highest muscle attenuation values also had the highest muscle quality. Almost half of the explained variance in muscle quality was due to attenuation values. The decline in muscle quality reported from the HABC study measured as knee extensor torque per unit total leg LTM is 12.1 and 10.1% per decade for men and women respectively (Newman et al. 2003). Given that the decline in total leg LTM, from cross-sectional analysis, was 9–10% per decade in the HABC study, it suggests a closer approximation between SM change and muscle quality change in those >70 y. Muscle quality (N m/cm^2^ or N m/kg) declines in 3 and 5-year longitudinal follow ups were as high as ~19–22% per decade in women and 26–27% in men (Goodpaster et al. 2006; Delmonico et al. 2009).

## Functional capability

The relative effort required to perform functional tasks increases with advancing age (Landers et al. 2001). Research designed to report the age-related decline in functional capability, require measures which can distinguish meaningful gradations of capacity and change over a wide range of abilities. Cohorts >50 y provide a challenge in the heterogeneity of their functional capabilities. Test batteries need to be able to reflect activities of daily living (ADL) and yet capture meaningful performance data relevant to the individual. As such there is a paucity of literature to report an estimate of the age-related decline in functional capability as we have done in the previous sections above. The SPPB (Guralnik et al. 1994) is the most commonly employed method of assessing the ability to perform ADL in mobility limited older adults. The battery uses a test of gait speed (6 m), lower extremity function (time taken to rise from a chair 5 times) and balance (semi-tandem and tandem stands) to make up a 12-point scoring system. The SPPB was validated in 5000 older adults (>71 y) and was found to predict nursing home admission. Since then many studies have used the SPPB to report older adult (>65 y) physical capability (Pahor et al. 2006; Vasunilashorn et al. 2009; Volpato et al. 2011). Furthermore, performance in this test battery or components of it have been associated with components of SM and muscle function discussed above.

Although 6–10 m gait speed tests may be considered highly representative of ADL they may suffer from either a floor or ceiling effect. In the case of the floor effect, a frail older adult may not be able to complete five chair rises and therefore cannot attain the minimum test score. Alternatively, in the case of the ceiling effect, the majority of physically active older adults may achieve the maximum test score, meaning the test cannot detect meaningful gradations of capacity and change over a wide range of abilities. For example, recent evidence from our group (Francis et al. 2017a) and Glenn et al. (2015) suggest that short (≤10 m) gait speed tests cannot detect change where expected in healthy older (50–70 y) and middle aged (55–64 y) adults respectively. This is largely due to the relative health of both cohorts indicated by a habitual gait speed (1.4 m/s) far in excess of the gait speed suggested to be indicative of disability (<0.8 m/s). Furthermore, the link between muscle mass, strength and functional capability using these tests may not be as strong in middle aged or healthy older adults compared to frail or mobility limited older adults. For example, Buchner et al. (1996) reported that the relationship between leg strength and gait speed (15.2 m) was non-linear. For stronger participants, there was no relationship between strength and gait speed but in weaker individuals there was. Therefore, small changes in physiological capacity of frail older adults may lead to large changes in functional capability whereas small changes in physiological capacity of strong adults may lead to no change in functional capability assessed in this way.

Tests which can allow participants perform to a greater maximum may be more appropriate to track age-related change in functional capacity prior to disablement. The 6 min walk test (Rikli and Jones 1998) and 30 s chair rise test (Jones et al. 1999) were originally designed to combat the floor effect for i.e. for participants who could not complete a full test e.g. five chair rises. However, the authors report the tests as being capable of detecting difference in functional capability between the 7th, 8th and 9th decade of life as well as performance differences between those with high and low self-reported physical activity. The construct validity of these tests is underlined by the fact that these data arise from normative data collected on 7183 community dwelling older (60–94 y) adults. Most recently, we reported the 30 s chair rise test and a 900 m extended gait speed test (Fig. 1) as capable of detecting change in functional capability between the 6th and 7th decade in healthy older adults (Francis et al. 2017a). Furthermore, knee extensor strength corrected for body mass and to a lesser extent muscle quality were associated with functional capability in healthy older women (Francis et al. 2017b). However, when both tests were used to assess the efficacy of a 12 week progressive resistance training intervention, only the 900 m gait speed test was responsive to the intervention (Francis et al. 2016a). The fact that our data and others (Hairi et al. 2010) have identified muscle strength (grip and quadriceps strength) as having stronger associations with functional capability than muscle quality may begin to question the functional significance of muscle quality as a measure in this context. This is a potentially important finding, if confirmed, given the considerable increase in time and expense to measure muscle quality relative to normalising strength to body mass. This does not discount muscle quality as an index as it may be important to understanding physiological changes at the tissue level.

Other test batteries often retain the core physical competencies assessed in the SPPB, whilst adding modifications in order to try and accommodate a broader range of abilities. The American Alliance for Health, Physical Education, Recreation & Dance (AAHPERD) Functional Fitness (Yaguchi and Furutani 1998) includes an extended gait speed test (880 yard walk). Outside of test batteries, the extended gait speed test (Simonsick et al. 2001) and the ten step stair climb power test (Bean et al. 2002) have also been deployed to measure functional capability. None of these tests however, have the normative data of those developed by Rikli and Jones. In order to report functional capability in healthy adults, specifically lower extremity functional capability across the lifespan, we recommend researchers select tests which allow participants to perform to a greater maximum. This would facilitate collection of meaningful performance data in conjunction with laboratory measures of SM and strength. This recommendation is based on studies which intend to measure healthy well-functioning adults that would not have trouble at least in walking for 6 min or 900 m and/or completing chair rises repeatedly for 30 s. In this population, extended tests may provide meaningful information on the relative effort required to go for a walk or spend a day in a town or city. These are activities which may be impaired prior to a reduction in the ability to complete basic tasks such as rising from a chair or walking 10 m and therefore may provide a more sensitive estimate of functional decline in healthy aging.

## Conclusion

In summary, based on the literature reviewed we recommend references ranges for LTM or SM are generated from the thigh region as this appears most responsive to age-related change or therapeutic intervention. The maximal force generating capacity of the thigh is best represented from the knee extensors due to the fact that it represents the majority of the thigh region, is a more stable measure, plays a greater role in activities of daily living and undergoes a preferential decline SM with ageing compared to the knee flexors. In light of these recommendations we also recommend that indices of muscle quality are generated using knee extensor torque per unit SM or LTM. There is a need for performance measures which can distinguish gradations of capacity and change over a wide range of abilities, therefore we recommend researchers use measures which allow performance to a greater maximum in order to simultaneously combat potential floor or ceiling effects.

## Abbreviations

CSA: Cross sectional area
SPPB: Short physical performance battery
MRI: Magnetic resonance imaging
CT: Computed tomography
SM: Skeletal muscle
DXA: Dual X-ray absorptiometry
LTM: Lean tissue mass
FFM: Fat free mass
BLSA: Baltimore longitudinal study of aging
HABC: Healthy aging and body composition
US: Ultrasound
BMI: Body mass index
ADL: Activities of daily living
AAHPERD: American Alliance for Health, Physical Education, Recreation & Dance
